# BDNF deficiency and young-adult methamphetamine induce sex-specific effects on prepulse inhibition regulation

**DOI:** 10.3389/fncel.2013.00092

**Published:** 2013-06-12

**Authors:** Elizabeth E. Manning, Maarten van den Buuse

**Affiliations:** ^1^Behavioural Neuroscience Laboratory, The Florey Institute of Neuroscience and Mental HealthMelbourne, VIC, Australia; ^2^Department of Pharmacology, The University of MelbourneMelbourne, VIC, Australia

**Keywords:** methamphetamine, prepulse inhibition, schizophrenia, BDNF

## Abstract

Brain-derived neurotrophic factor (BDNF) has been implicated in the pathophysiology of schizophrenia, yet its role in the development of specific symptoms is unclear. Methamphetamine (METH) users have an increased risk of psychosis and schizophrenia, and METH-treated animals have been used extensively as a model to study the positive symptoms of schizophrenia. We investigated whether METH treatment in BDNF heterozygous (HET) mutant mice has cumulative effects on sensorimotor gating, including the disruptive effects of psychotropic drugs. BDNF HETs and wildtype (WT) littermates were treated during young adulthood with METH and, following a 2-week break, prepulse inhibition (PPI) was examined. At baseline, BDNF HETs showed reduced PPI compared to WT mice irrespective of METH pre-treatment. An acute challenge with amphetamine (AMPH) disrupted PPI but male BDNF HETs were more sensitive to this effect, irrespective of METH pre-treatment. In contrast, female mice treated with METH were less sensitive to the disruptive effects of AMPH, and there were no effects of BDNF genotype. Similar changes were not observed in the response to an acute apomorphine (APO) or MK-801 challenge. These results show that genetically-induced reduction of BDNF caused changes in a behavioral endophenotype relevant to the positive symptoms of schizophrenia. However, major sex differences were observed in the effects of a psychotropic drug challenge on this behavior. These findings suggest sex differences in the effects of BDNF depletion and METH treatment on the monoamine signaling pathways that regulate PPI. Given that these same pathways are thought to contribute to the expression of positive symptoms in schizophrenia, this work suggests that there may be significant sex differences in the pathophysiology underlying these symptoms. Elucidating these sex differences may be important for our understanding of the neurobiology of schizophrenia and developing better treatments strategies for the disorder.

## Introduction

Schizophrenia is a debilitating neuropsychiatric disease, and despite numerous factors being associated with disease risk and pathophysiology, the etiology of the illness is poorly understood. This gap in our knowledge has hindered the development of new treatment strategies that target the positive, negative and cognitive symptoms on the illness. Altered brain-derived neurotrophic factor (BDNF) signaling has been associated with schizophrenia through post-mortem, blood biomarker and genetic association studies (Angelucci et al., 2005). Post-mortem studies have consistently shown reduced BDNF expression in the hippocampus and dorso-lateral prefrontal cortex (DLPFC) of patients with schizophrenia, two brain regions that are highly implicated in the pathophysiology of the disorder (Takahashi et al., 2000; Weickert et al., 2003; Hashimoto et al., 2005; Wong et al., 2010; Thompson Ray et al., 2011). These findings have lead to numerous studies aiming to asses peripheral BDNF as a biomarker for the illness, and a recent meta-analysis found a moderate decrease in blood BDNF levels in drug-naïve and medicated schizophrenia patients (Green et al., 2011). Others have shown an association between the BDNF gene single nucleotide polymorphism (SNP) *val66met* and schizophrenia in some populations (Neves-Pereira et al., 2005; Gratacos et al., 2007). Although this has not been replicated in other population and genome-wide association studies, other groups have shown associations in patients with schizophrenia between the SNP and age of onset (Numata et al., 2006; Chao et al., 2008; Zhou et al., 2010), cognitive performance (Ho et al., 2006; Rybakowski et al., 2006; Kebir et al., 2009; Lu et al., 2012), and neuroimaging measures (Szeszko et al., 2005; Koolschijn et al., 2010; Smith et al., 2012). Interestingly, there are major sex-differences in the age of onset of schizophrenia and also symptom severity, with males showing earlier onset of disease and greater cognitive deficits (Hafner et al., 1993; Goldstein et al., 1998). It has recently been proposed that altered BDNF signaling in the disorder may contribute to these sex differences (Hill, 2012).

BDNF is important for neuronal differentiation and survival during early brain development, and has further roles in synaptic plasticity in the adult brain, however it is unclear how altered BDNF signaling may contribute to disruption of behavior related to the symptoms observed in schizophrenia. BDNF knock-out mice have a severe phenotype and typically do not survive beyond 3 weeks of age, whereas the BDNF heterozygous (HET) mice exhibit a more subtle phenotype and normal survival. We have previously shown that BDNF protein levels are reduced by a third to a half of WT expression levels throughout the brain in BDNF HETs (Hill and van den Buuse, 2011). This is similar to the reduction of BDNF protein observed in schizophrenia (Weickert et al., 2003), therefore these animals can be used as a model to examine the role of BDNF in behaviors related to schizophrenia. Indeed, this strain has been extensively characterized during the last decade in behavioral endophenotypes related to psychiatric disorders, with most studies showing little disturbance in baseline behavior, making them appropriate for experiments examining the effects of additional environmental manipulations. We have recently shown that male BDNF HETs show an increased vulnerability to the effects of young-adult corticosterone treatment, which caused a deficit in short-term spatial memory in a Y-maze task with parallel alterations in hippocampal NMDA receptor subunit expression (Klug et al., 2012). Others have demonstrated that BDNF HETs show altered sensitivity to the effects of stress and antidepressant treatments in paradigms testing anxiety and depressive-like behavior (Saarelainen et al., 2003; Duman et al., 2007, 2008; Ibarguen-Vargas et al., 2009). These findings support the notion that reduced BDNF expression, as is observed in schizophrenia, may alter an individual's sensitivity to the effects of other environmental risk factors (Caspi and Moffitt, 2006).

Drug abuse is one environmental factor that has been associated with increased risk of neuropsychiatric illnesses (Gururajan et al., 2012), and methamphetamine (METH) users have an increased risk of psychosis and schizophrenia (McKetin et al., 2010; Callaghan et al., 2012). While not all users will experience long-lasting psychosis, those who do tend to have an earlier age of first METH use, and increased familial risk of schizophrenia (Chen et al., 2003, 2005). These findings suggest that young adulthood may be a period of particular vulnerability, and that genetic factors also contribute to an individual's risk. One group has shown that the BDNF *val66met* SNP is associated with psychosis in Chinese METH users but not those from other ethnicities (Sim et al., 2010). Interestingly, this study found that METH dependence was associated with the *val* allele, while psychosis in METH users was associated with the *met* allele that has previously been linked with schizophrenia risk and disease outcomes. In animal models, BDNF expression has been shown to change following acute and repeated treatment with amphetamines (Le Foll et al., 2005; Angelucci et al., 2007), and rodent models with genetically modified expression of BDNF, including BDNF HETs, show altered sensitivity to a number of stimulant drugs (Hall et al., 2003; Bahi et al., 2008; Saylor and McGinty, 2008).

The aim of the current study was to examine the long-term effects of chronic METH treatment in BDNF HETs during young-adulthood on a behavioral endophenotype related to the positive symptoms of schizophrenia, prepulse inhibition (PPI) of the acoustic startle reflex. We predicted that METH administration in an escalating dosing regime during young adulthood would cause a disruption of PPI in adulthood, and given that BDNF has been implicated in the actions of METH previously, that BDNF HETs may respond to this treatment differentially. Given that BDNF levels are reduced in schizophrenia, BDNF HETs may be more likely to show a disturbance of PPI following METH exposure. PPI is a measure of sensorimotor gating, a form of information processing that is disrupted in patients with schizophrenia. Reduced PPI in schizophrenia is associated with thought disorder and functional impairment (Perry and Braff, 1994; Swerdlow et al., 2006), and deficits in PPI are considered to be a valid endophenotype related to the positive symptoms of the illness. PPI is one of the most widely used behavioral tests in mouse models related to schizophrenia, due to its high construct validity across species compared to other behavioral measures relevant to the disorder (Powell et al., 2009). The neuropharmacology of PPI has been characterized extensively in humans and rodent models, implicating dopaminergic, serotonergic, and glutamatergic signaling pathways (Geyer et al., 2001). Acute challenge with psychotropic drugs that target these neurotransmitter systems causes a disruption of PPI similar to what is observed in schizophrenia (van den Buuse, 2010). Our laboratory and others routinely use the psychotropic drugs amphetamine (AMPH), apomorphine (APO), and MK-801 to investigate the neuropharmacology of behavioral endophenotypes relevant to the positive symptoms of schizophrenia (Chavez et al., 2009). Acute AMPH can mimic behavioral changes observed in schizophrenia, largely due to increasing subcortical release of dopamine, similar to what is thought to cause psychotic episodes in schizophrenia (Abi-Dargham et al., 1998). Challenge with the D1/D2 receptor agonist APO can also be used to further investigate dopaminergic regulation of PPI. In contrast, the NMDA receptor antagonist MK-801 is used as an acute drug challenge model of the “glutamate hypofunction” hypothesis of schizophrenia (Lahti et al., 1995). By examining PPI during adulthood at baseline and following challenge with AMPH, APO and MK-801, we aimed to understand how BDNF depletion and METH treatment during young adulthood may interact to affect the regulation of this information processing mechanism, and whether neurotransmitter systems related to the pathophysiology of schizophrenia may be involved in their effects.

## Results

### Effects of chronic METH treatment on body weight and locomotor activity do not differ between BDNF HETs and WT littermates

**Figure 1:**
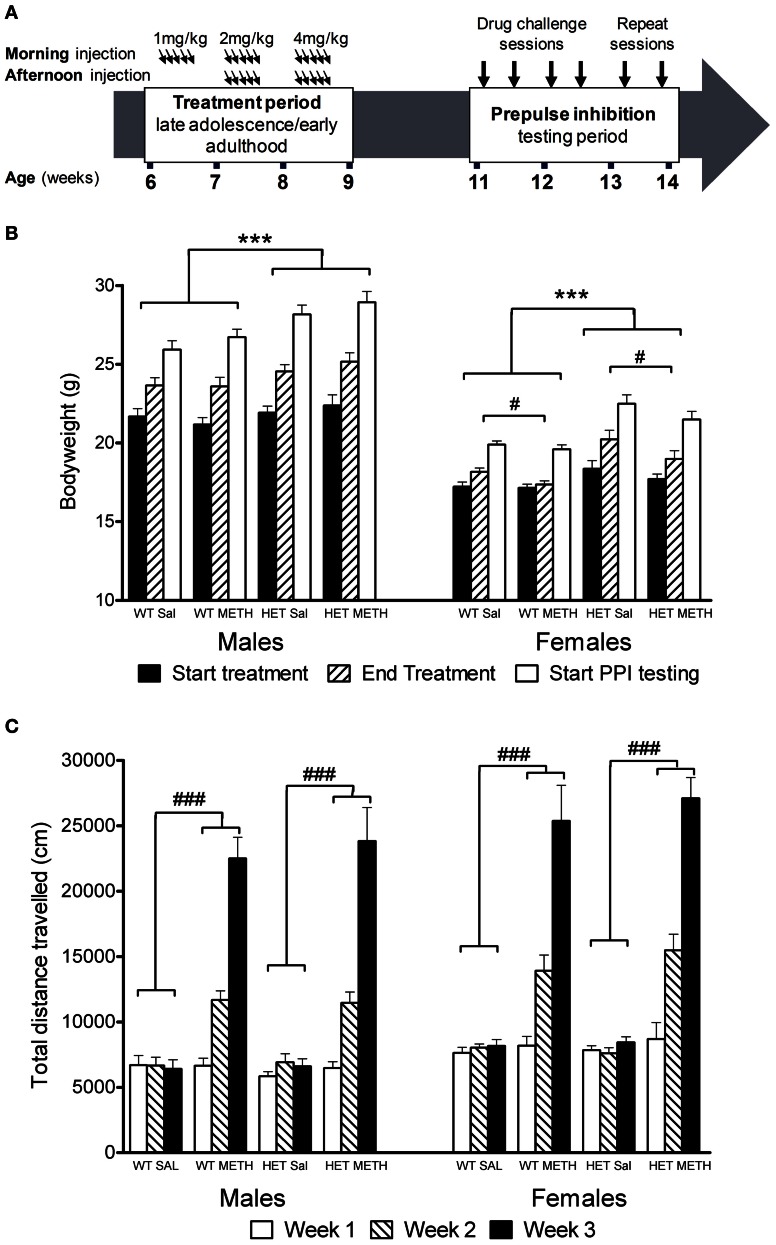
**Experimental timeline, bodyweight change, and locomotor activity during the treatment period. (A)** Mice were treated with saline or an escalating dosing regime of METH from 6 to 9 weeks of age, i.e., during late adolescence/early adulthood. Following a 2-week break, the animals were tested in four PPI sessions following challenge treatment with either saline, AMPH, APO, or MK-801 in pseudorandomized order. **(B)** Bodyweight at the start or end of the chronic METH/saline treatment and at the start of PPI testing. BDNF HETs were significantly heavier than their WT littermates throughout the experiment. At the end of the chronic treatment period, METH-treated females had reduced bodyweight compared to saline-treated mice. **(C)** Locomotor activity recorded at the end of each subsequent treatment week following morning injections. METH-treated mice showed significant hyperactivity compared to saline-treated mice, which increased during the treatment period with increasing doses of METH administration. This effect did not differ between the genotypes. ^***^Signifies genotype effect *p* < 0.001, ^#^signifies METH × time-point interaction *p* < 0.05, ^###^signifies METH × week interaction *p* < 0.001.

BDNF HETs of both sexes were heavier than their wildtype (WT) littermates throughout the treatment and behavioral testing periods [Figure 1B, main effect genotype *F*_(1, 66)_ = 25.6, *p* < 0.001]. In addition, during the treatment period female mice treated with METH did not show the same bodyweight increase as females treated with saline [first day of treatment vs. last day of treatment: sex × METH treatment × time-point interaction: *F*_(1, 66)_ = 4.9, *p* = 0.031; females only: METH treatment × time-point *F*_(1, 33)_ = 5.7, *p* = 0.023]. METH-treated female mice showed significant recovery of bodyweight gain during the weeks following the end of the treatment period despite their bodyweight remaining lower than saline-treated female mice during the remainder of the experimental period [weekly bodyweight in the 5 weeks following the end of the treatment period, sex × METH treatment interaction *F*_(1, 66)_ = 4.6, *p* = 0.037]. There was no body weight gain delay in male BDNF HETs. Importantly, METH treatment did not result in bodyweight *loss* in any of the groups.

METH administration during the 3-week treatment period caused significant hyperactivity [Figure 1C, main effect of METH: *F*_(1, 53)_ = 208.0, *p* < 0.001] which became more prominent over the escalating dosing period [METH × treatment week interaction: *F*_(2, 106)_ = 189.7, *p* < 0.001]. At the end of the first treatment week, there was no effect of the 1 mg/kg dose on locomotor activity. Subsequently, however, the animals showed increasing levels of hyperactivity in response to the higher doses of METH administered during the second and third weeks of the treatment period. There were no differences between male and female mice or between BDNF HETs and WT mice in the extent of METH-induced hyperactivity during the treatment period (Figure 1C).

### Reduced baseline PPI and increased startle reactivity in BDNF HETs

**Figure 2:**
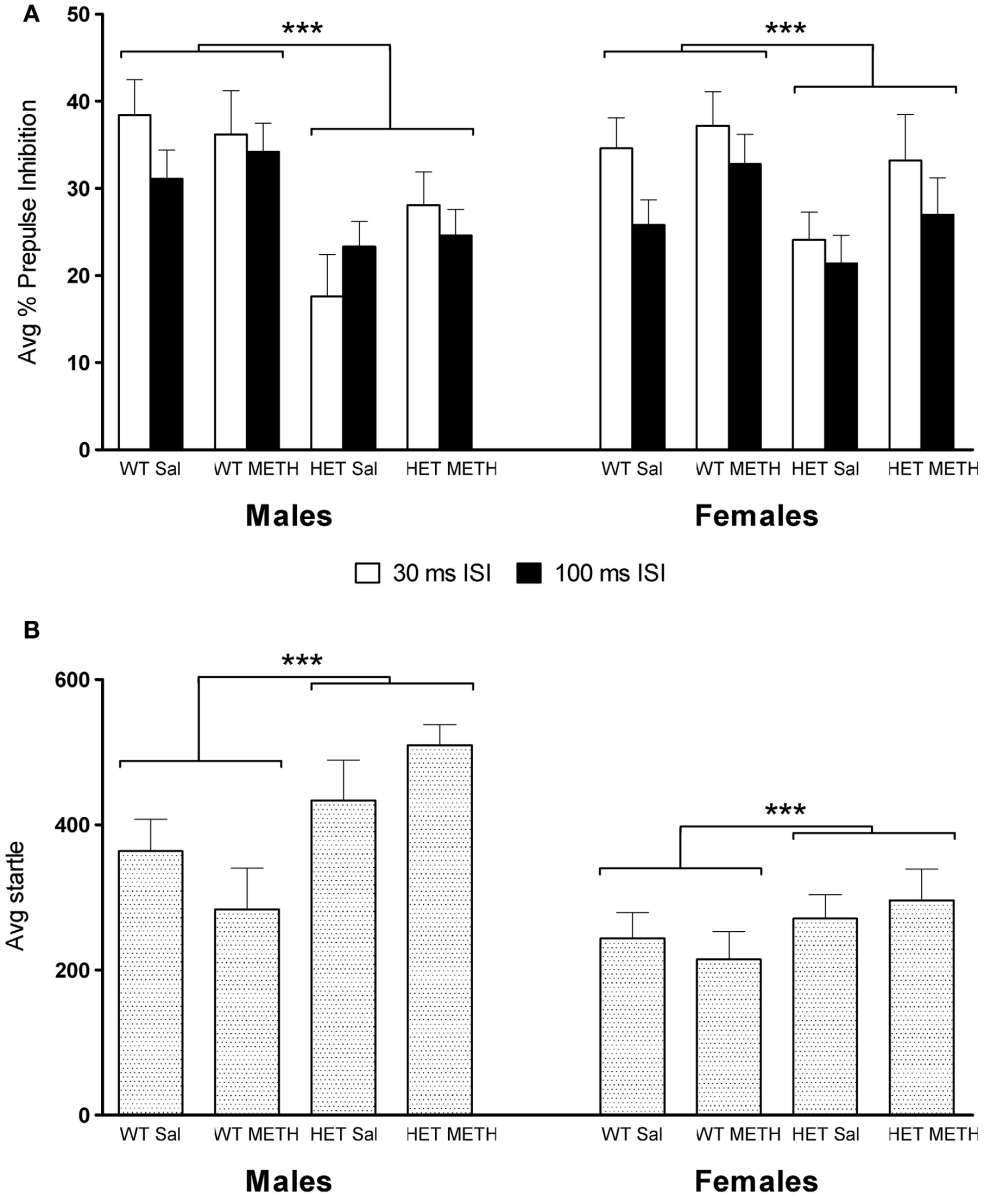
**PPI and startle reflex at baseline during saline challenge sessions. (A)** Average PPI across all four PP intensities was significantly lower in BDNF HETs compared to WT controls. This effect was seen at both the 30 and 100 ms ISI and in both sexes. **(B)** Average startle responses across all four startle blocks were significantly increased in BDNF HETs of both sexes compared to WT controls. ^***^Signifies genotype effect *p* < 0.005.

Baseline PPI, obtained after injection of saline, was significantly lower in BDNF HET mice compared to WT [Figure 2A, main effect of genotype at 30 ms inter-stimulus interval (ISI) *F*_(1, 66)_ = 13.1, *p* = 0.001; 100 ms ISI: *F*_(1, 66)_ = 8.8, *p* = 0.004]. This effect was independent of METH treatment or the sex of the animals (Figure 2A).

Average startle across the saline session was significantly higher in male mice compared to female mice [Figure 2B, main effect of sex: *F*_(1, 66)_ = 21.6, *p*<0.001]. In addition, baseline startle was significantly higher in BDNF HETs compared to WT controls [main effect of genotype *F*_(1, 66)_ = 11.1, *p* = 0.001]. In male mice, but not female mice, habituation of startle across the 4 blocks of stimulus-only pulses was slightly accelerated in BDNF HETs compared to WT controls [data not shown; startle block × genotype × sex interaction: *F*_(3, 198)_ = 4.7, *p* = 0.005].

### Sex-dependent changes in the effect of AMPH on PPI and startle reactivity

**Figure 3:**
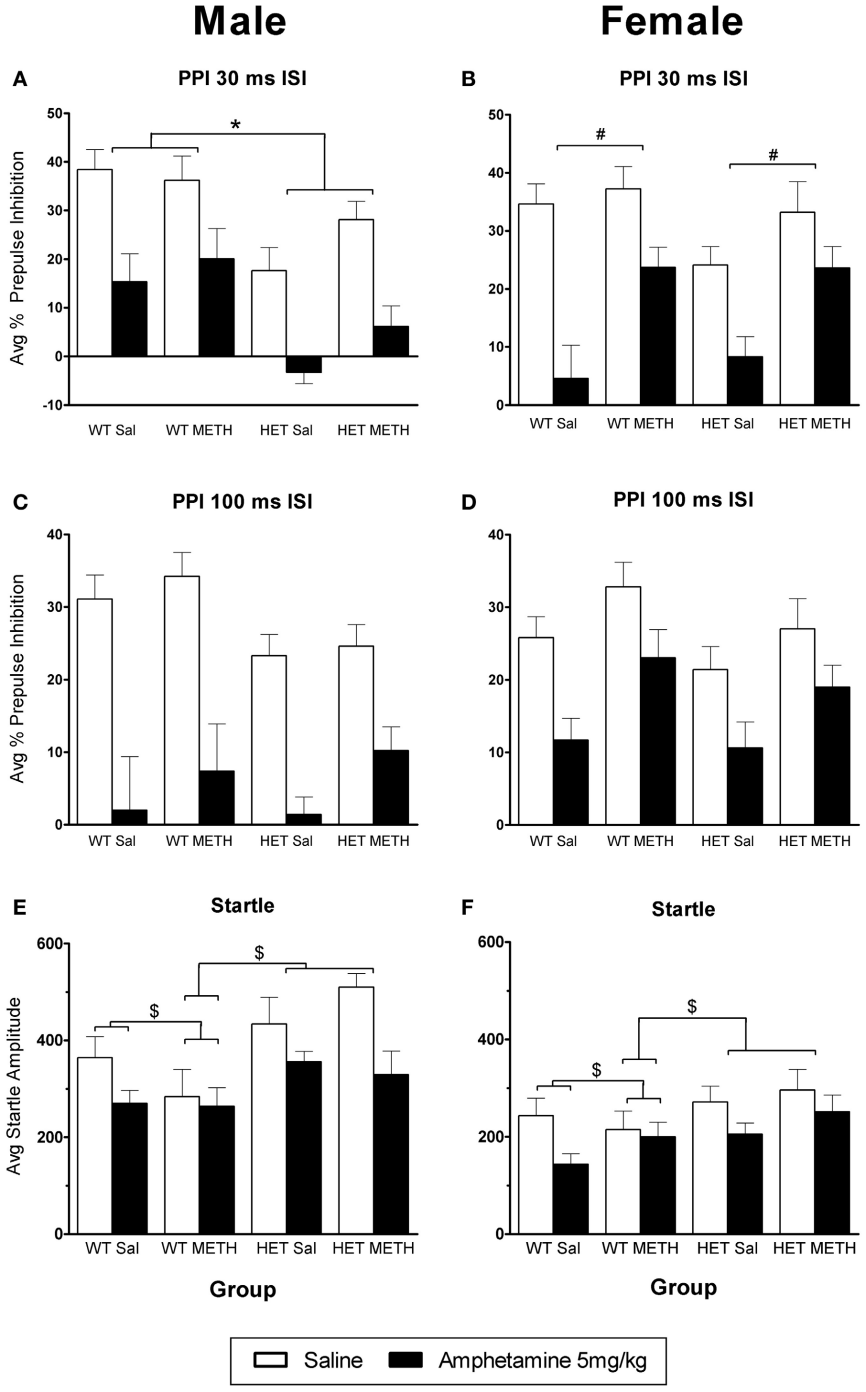
**AMPH-induced disruption of PPI. (A,B)** At the 30 ms ISI, following an AMPH challenge, PPI was disrupted in all groups, however the effect of AMPH was enhanced in male BDNF HETs **(A)**, and was attenuated in female mice treated with METH **(B)**. **(C,D)** At the 100 ms ISI, following an AMPH challenge PPI was disrupted in all groups, however this was not significantly different between the treatment groups. **(E,F)** Startle responses following AMPH challenge were reduced, however this effect was not seen in WT mice treated with METH. ^*^Signifies genotype × AMPH × PP interaction *p* < 0.05, ^#^signifies METH treatment × AMPH interaction *p* < 0.05, ^$^signifies AMPH × genotype × METH treatment interaction *p* < 0.031.

AMPH administration caused a significant disruption of PPI at both ISIs [Figures 3A–D, main effect, 30 ms ISI: *F*_(1, 66)_ = 124.0, *p* < 0.001, 100 ms ISI: *F*_(1, 66)_ = 87.8, *p* < 0.001]. However, at the 30ms ISI, there were significant sex-dependent effects of genotype and METH treatment on the response to AMPH challenge [AMPH × genotype × sex × prepulse (PP) interaction: *F*_(3, 198)_ = 3.5, *p* = 0.026] and further analysis was therefore done on the data separated by the sex of the animals. In male BDNF HET, irrespective of METH pre-treatment, PPI was disrupted to a greater extent by an acute AMPH challenge than in male WT, particularly at higher PP intensities [Figure 3A, AMPH × genotype × PP interaction: *F*_(3, 99)_ = 4.3, *p* = 0.013, see also Table 1 for PPI at each PP intensity]. In contrast, female mice treated with METH showed a reduced sensitivity to the disruptive effects of an acute AMPH challenge, irrespective of the genotype [Figure 3B, interaction of AMPH × METH treatment: *F*_(1, 33)_ = 4.6, *p* = 0.039, see also Table 2 for PPI at each PP intensity]. At the 100 ms ISI, there was a strong trend for reduced sensitivity to the disruptive effects of AMPH in METH-treated mice of both sexes (Figures 3C,D, AMPH × METH treatment interaction *p* = 0.063).

**Table 1:** **Mean PPI ± SEM at each PP intensity during challenge sessions with saline, AMPH, APO or MK-801 treatment in male WT and BDNF HET mice chronically pre-treated with saline or METH**.

	**30 ms ISI**					**100 ms ISI**				
	**PP2**	**PP4**	**PP8**	**PP16**	**Average**	**PP2**	**PP4**	**PP8**	**PP16**	**Average**
**MALE WT SALINE**										
Saline	19.7 ± 7.5	21.4 ± 7.1	44.9 ± 5.5	67.6 ± 3.9	38.4 ± 4.1	24.8 ± 5.0	25.4 ± 4.9	23.9 ± 4.0	50.5 ± 3.8	31.1 ± 3.3
AMPH	−15.5 ± 8.8	1.5 ± 7.6	24.9 ±7.6	50.5 ± 5.1	15.3 ± 5.8	−4.9 ± 9.1	−2.7 ± 4.6	12.2 ± 3.8	31.4 ± 4.2	2.0 ± 7.4
APO	−28.8 ± 10.5	1.9 ± 5.7	22.8 ± 8.4	46.3 ± 4.9	10.5 ± 4.6	1.5 ± 3.3	−4.2 ± 6.3	13.1 ± 3.3	34.9 ± 4.3	11.3 ± 2.8
MK-801	0.5 ± 7.9	6.7 ± 4.7	23.7 ± 5.3	45.1 ± 4.4	19.0 ± 3.8	−5.6 ± 7.3	−15.4 ± 14.9	−2.4 ± 10.1	31.4 ± 4.2	2.0 ± 7.4
**MALE WT METH**										
Saline	5.1 ± 10.5	23.5 ± 5.9	44.7 ± 5.6	71.7 ± 2.4	36.2 ± 5.0	14.0 ± 6.3	27.9 ± 4.4	37.5 ± 4.1	57.6 ± 4.4	34.2 ± 3.3
AMPH	−20.4 ± 13.7	1.0 ± 10.6	38.1 ± 7.8	61.4 ± 4.6	20.0 ± 6.3	−10.8 ± 8.9	6.6 ± 7.5	28.1 ± 5.7	31.8 ± 6.4	7.4 ± 6.5
APO	−13.2 ± 12.7	2.5 ± 10.2	24.6 ± 12.6	45.3 ± 11.6	14.8 ± 10.2	−3.6 ± 8.1	4.4 ± 8.6	31.9 ± 8.1	41.1 ± 4.4	18.5 ± 5.8
MK-801	0.6 ± 8.8	15.4 ± 8.5	27.5 ± 7.8	42.6 ± 11.8	21.5 ± 8.2	−8.2 ± 8.5	−6.9 ± 10.2	12.9 ± 9.1	31.8 ± 6.4	7.4 ± 6.5
**MALE BDNF HET SALINE**										
Saline	4.6 ± 6.6	−1.6 ± 7.4	19.3 ± 6.5	48.0 ± 5.2	17.6 ± 4.8	15.5 ± 3.6	15.7 ± 2.9	20.5 ± 4.9	41.4 ± 6.2	23.3 ± 2.9
AMPH	−18.4 ± 7.5	−18.1 ± 4.4	−1.6 ± 6.4	24.9 ± 8.2	−3.3 ± 2.3	−8.8 ± 4.9	−9.1 ± 7.8	4.1 ± 5.2	19.7 ± 3.9	1.4 ± 2.4
APO	−1.4 ± 5.8	−6.8 ± 8.1	4.3 ± 10.0	21.4 ± 8.4	4.4 ± 6.9	0.7 ± 3.1	−2.5 ± 5.1	12.0 ± 3.6	21.1 ± 5.1	7.8 ± 3.5
MK-801	4.9 ± 6.1	3.1 ± 4.3	9.9 ± 5.5	35.2 ± 6.7	13.3 ± 3.8	−5.9 ± 8.5	3.9 ± 5.6	4.8 ± 9.4	28.3 ± 4.5	7.8 ± 5.1
**MALE BDNF HET METH**										
Saline	−3.5 ± 9.5	25.5 ± 4.9	32.2 ± 5.9	58.4 ± 4.4	28.1 ± 3.8	14.5 ± 3.3	23.3 ± 3.9	21.2 ± 4.1	39.3 ± 4.1	24.6 ± 3.0
AMPH	−7.3 ± 5.1	−0.9 ± 6.3	−4.2 ± 6.9	36.7 ± 6.5	6.1 ± 4.3	0.0 ± 3.3	3.1 ± 4.0	6.9 ± 5.7	30.9 ± 4.3	10.2 ± 3.3
APO	−14.8 ± 9.9	−3.7 ± 6.6	−0.5 ± 12.7	30.7 ± 5.2	2.9 ± 6.0	−5.1 ± 8.4	−6.5 ± 7.2	9.9 ± 6.6	30.8 ± 7.1	7.3 ± 4.5
MK-801	1.2 ± 7.0	−8.2 ± 8.0	4.9 ± 10.7	39.1 ± 5.8	9.3 ± 6.1	−24.2 ± 10.8	−17.5 ± 6.6	−0.2 ± 9.8	25.8 ± 6.4	−4.0 ± 6.5

**Table 2:** **Mean PPI ± SEM at each PP intensity during challenge sessions with saline, AMPH, APO, or MK-801 treatment in female WT and BDNF HET mice chronically pre-treated with saline or METH**.

	**30 ms ISI**					**100 ms ISI**				
	**PP2**	**PP4**	**PP8**	**PP16**	**Average**	**PP2**	**PP4**	**PP8**	**PP16**	**Average**
**FEMALE WT SALINE**										
Saline	7.8 ± 7.8	28.8 ± 3.7	39.7 ± 5.8	62.0 ± 3.3	34.6 ± 3.5	6.3 ± 7.4	16.2 ± 4.3	31.1 ± 5.1	49.5 ± 3.6	25.8 ± 2.9
AMPH	−12.7 ± 7.3	−6.6 ± 5.0	8.5 ± 8.2	29.2 ± 7.6	4.6 ± 5.7	−7.1 ± 6.2	−4.4 ± 7.9	10.4 ± 8.6	27.8 ± 5.5	11.7 ± 3.0
APO	−17.1 ± 8.7	−9.5 ± 8.7	18.3 ± 8.8	33.0 ± 5.5	6.2 ± 5.5	−12.6 ± 3.6	2.2 ± 5.9	15.0 ± 5.7	25.5 ± 7.7	7.5 ± 4.1
MK-801	−1.0 ± 4.6	14.4 ± 3.7	16.3 ± 6.2	43.7 ± 5.6	18.4 ± 4.0	−3.5 ± 5.3	9.1 ± 3.3	13.5 ± 4.1	27.8 ± 5.5	11.7 ± 3.0
**FEMALE WT METH**										
Saline	18.4 ± 7.3	27.5 ± 4.5	39.8 ± 5.3	63.2 ± 2.6	37.2 ± 3.9	18.4 ± 4.7	26.8 ± 4.9	35.7 ± 3.5	50.1 ± 4.0	32.8 ± 3.4
AMPH	3.3 ± 6.4	5.9 ± 5.3	37.6 ± 4.2	48.0 ± 3.4	23.7 ± 3.5	3.5 ± 4.2	15.5 ± 5.4	25.3 ± 6.4	47.8 ± 4.5	23.0 ± 3.9
APO	5.4 ± 6.0	9.8 ± 8.1	26.5 ± 7.5	44.2 ± 6.3	21.5 ± 5.4	−3.5 ± 7.7	9.5 ± 3.2	16.9 ± 6.5	38.1 ± 4.7	15.3 ± 3.9
MK-801	11.5 ± 5.3	14.6 ± 6.4	29.8 ±5.4	50.4 ± 3.9	26.6 ± 3.8	3.0 ± 6.1	7.5 ± 4.0	24.9 ± 6.2	43.0 ± 5.2	19.6 ± 4.2
**FEMALE BDNF HET SALINE**										
Saline	13.3 ± 6.3	16.1 ± 4.4	26.0 ± 3.2	40.9 ± 4.9	24.1 ± 3.2	8.3 ± 4.9	18.9 ± 4.5	23.6 ± 3.9	34.8 ± 4.5	21.4 ± 3.2
AMPH	−3.5 ± 3.9	−0.1 ± 6.0	5.2 ± 6.3	31.6 ± 4.4	8.3 ± 3.5	5.5 ±3.8	1.5 ± 6.8	8.4 ± 4.4	26.9 ± 3.0	10.6 ± 3.6
APO	−25.4 ± 5.7	−12.4 ± 7.0	1.1 ± 8.2	20.2 ± 9.0	−4.1 ± 5.7	−15.0 ± 5.0	2.3 ± 6.7	10.1 ± 7.1	16.9 ± 6.7	3.6 ± 4.1
MK-801	0.4 ± 5.3	12.3 ± 7.6	26.4 ± 6.2	33.9 ± 8.6	18.2 ± 4.6	7.9 ± 3.6	5.5 ± 9.7	7.0 ± 9.2	28.3 ± 6.9	12.2 ± 6.0
**FEMALE BDNF HET METH**										
Saline	21.0 ± 9.5	23.7 ± 8.6	29.4 ± 4.4	58.5 ± 4.0	33.2 ± 5.3	17.0 ± 6.8	19.8 ± 4.7	29.8 ± 3.9	41.4 ± 5.0	27.0 ± 4.2
AMPH	3.7 ± 5.3	16.9 ± 8.2	28.1 ± 5.0	45.7 ± 5.6	23.6 ± 3.7	8.7 ± 4.1	7.9 ± 4.5	20.5 ± 4.8	38.8 ± 4.1	19.0 ± 3.0
APO	−8.1 ± 7.9	−6.8 ± 14.5	6.2 ± 8.5	35.0 ± 6.1	6.6 ± 8.2	−11.4 ± 8.5	1.8 ± 7.6	0.6 ± 5.6	20.8 ± 6.6	3.0 ± 6.0
MK-801	8.5 ± 8.5	7.8 ± 8.0	25.5 ± 5.9	44.3 ± 6.2	21.6 ± 5.5	5.5 ± 7.8	4.1 ± 6.5	20.1 ± 4.8	30.4 ± 6.2	15.0 ± 4.7

AMPH administration caused a reduction in startle amplitude compared to saline [Figures 3E,F, *F*_(1, 66)_ = 29.9, *p* < 0.001]. However, this effect of AMPH was reduced by prior METH treatment in a genotype-specific manner [AMPH × genotype × METH treatment interaction *F*_(1, 66)_ = 4.8, *p* = 0.031]. Irrespective of the sex of the animals, WT mice treated with METH did not show a reduction of startle in response to acute AMPH [WT only: AMPH × METH treatment interaction, *F*_(1, 36)_ = 4.5, *p* = 0.042]. This effect of METH pre-treatment was not observed in BDNF HETs.

### No major changes in the effects of APO in BDNF HETs or following METH treatment

**Figure 4:**
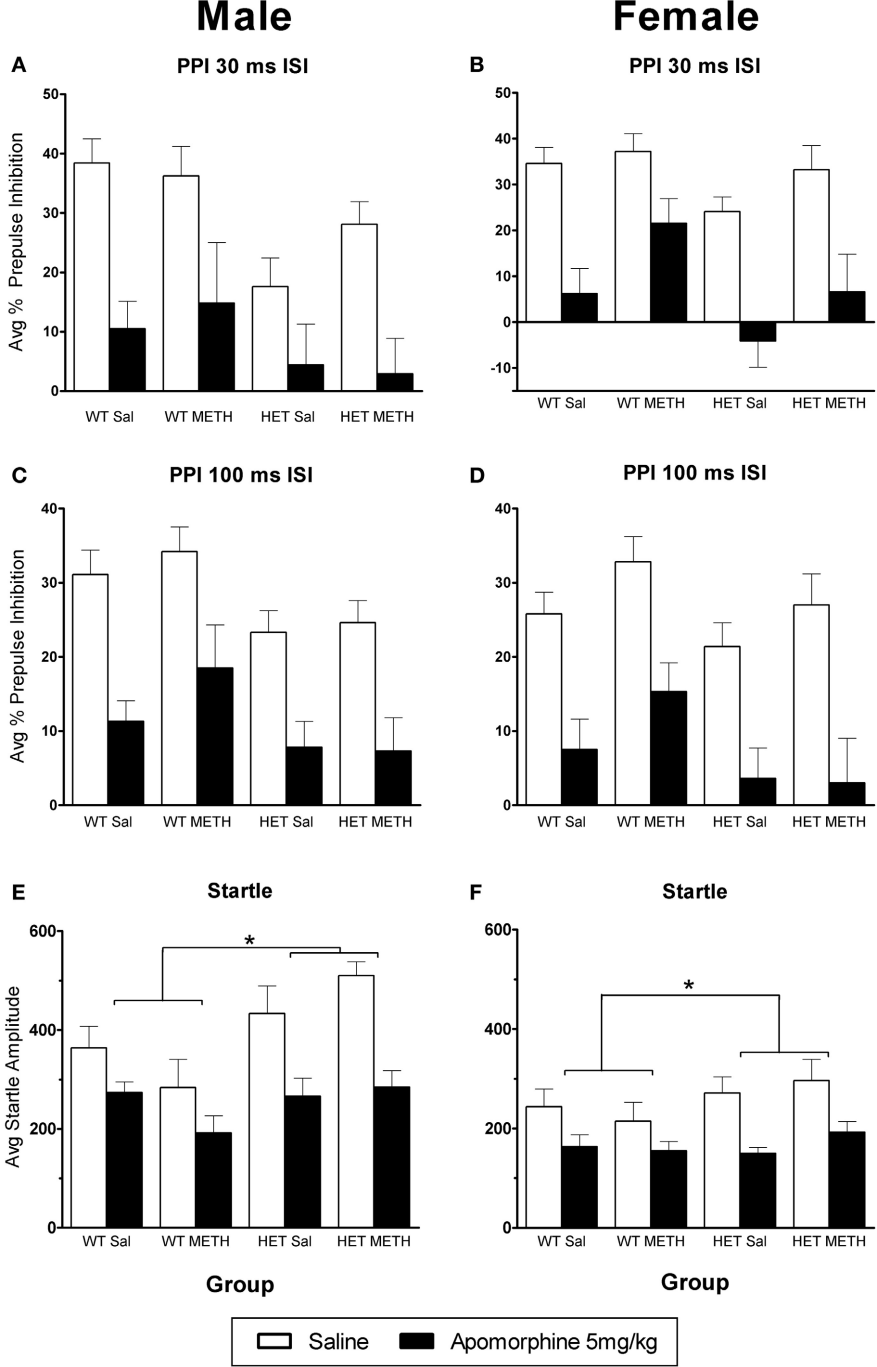
**APO-induced disruption of PPI. (A–D)** PPI following an APO challenge in male **(A)** and female **(B)** mice at the 30 ms ISI and male **(C)** and female **(D)** mice at the 100 ms ISI. APO disrupted PPI in all groups, and this was unaffected by BDNF genotype or METH treatment. **(E,F)** Startle responses following an APO challenge were reduced; however this effect was greater in BDNF HETs. *Signifies genotype × APO interaction *p* < 0.05.

Given that the major effects of AMPH on PPI in mice are thought to be due to indirect activation of dopaminergic receptors, we were interested to examine whether the D1/D2 receptor agonist APO would show similar effects to AMPH. APO caused a significant disruption of PPI [Figures 4A–D, at both ISIs: main effect of APO challenge: *F*_(1, 66)_> 85.0, *p* < 0.001]. In contrast to the sex-dependent effects of genotype and METH treatment that were observed in the sensitivity to AMPH, similar effects were not observed in the response to APO. A minor, but significant change in APO sensitivity was found as a PP-dependent genotype effect in male mice, where BDNF HETs were slightly less sensitive to the effects of APO, but only at the lowest PP intensity examined [30 ms ISI: APO challenge × genotype × sex × PP interaction *F*_(3, 198)_ = 3.1, *p* = 0.037; Table 1, PP2dB only: APO challenge × genotype × sex interaction *F*_(1, 66)_ = 5.0, *p* = 0.029].

APO also caused a significant reduction in startle response amplitude (Figures 4E,F). However, this effect was significantly larger in BDNF HETs [main effect of APO challenge: *F*_(1, 66)_ = 65.9, *p* < 0.001; APO × genotype interaction: *F*_(1, 66)_ = 6.5, *p* = 0.013], and this effect acted to normalize their startle response to that of WT animals treated with APO (APO only: main effect genotype *p* = 0.154).

### No major changes in the effects of MK-801 in BDNF HETs or following METH treatment

**Figure 5:**
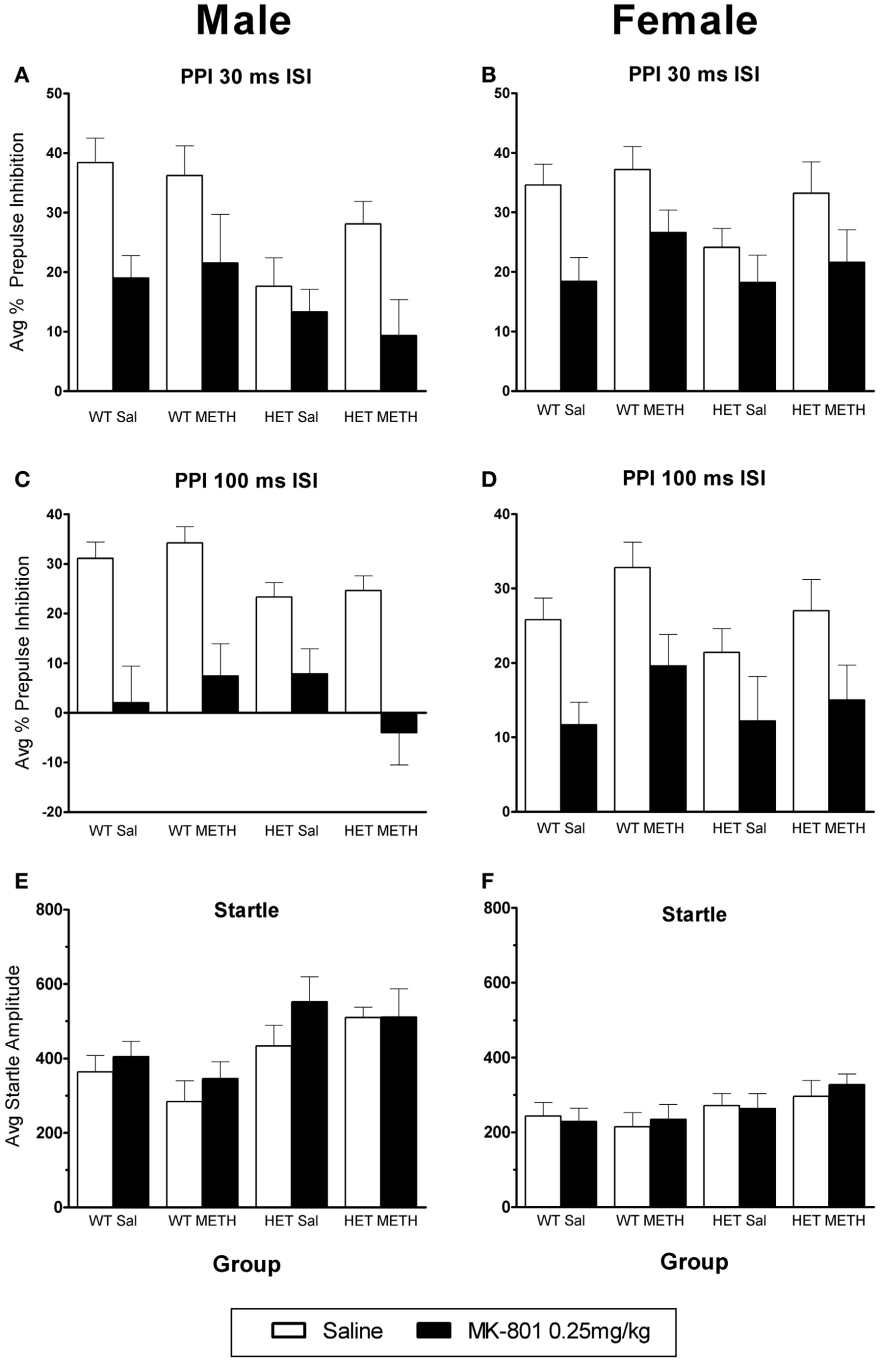
**MK-801-induced disruption of PPI. (A–D)** PPI following an MK-801 challenge in male **(A)** and female **(B)** mice at the 30 ms ISI and male **(C)** and female **(D)** mice at the 100 ms ISI. MK-801 disrupted PPI in all groups, and this was unaffected by BDNF genotype or METH treatment. **(E,F)** Startle response following an MK-801 challenge. There was a trend toward increased startle reactivity following MK-801 challenge (*p* = 0.081).

MK-801 caused a significant disruption of PPI [at both ISIs: main effect of MK-801: *F*_(1, 66)_ > 38.0, *p* < 0.001]. There were no significant changes in the response to MK-801 in BDNF HETs or following METH treatment (Figures 5A–D). There was no significant effect of MK-801 administration on startle amplitudes (Figures 5E,F).

## Discussion

The key findings of the current study were that (1) compared to WT controls, BDNF HETs showed reduced baseline PPI, irrespective of METH pre-treatment or the sex of the animals; (2) male BDNF HETs showed greater sensitivity to the disruptive effects of an AMPH challenge on PPI at the 30 ms ISI, irrespective of whether they had been pre-treated with METH; (3) female mice pre-treated with METH showed a relative tolerance to the effects of AMPH on PPI at the 30 ms ISI, irrespective of their genotype. Thus, there were no interactions between METH treatment and BDNF depletion on PPI, and no changes were observed in the PPI-disrupting effects of APO and MK-801 in this model. Startle response amplitude was higher in BDNF HETs compared to WT controls, which may be in part due to their increased bodyweight. AMPH and APO reduced startle reactivity, and there were significant genotype effects. METH treatment attenuated the effects of AMPH on startle in WT mice but not BDNF HETs, while the effects of APO on startle amplitude were greater in BDNF HETs.

The main aim of the present study was to investigate whether BDNF deficiency, as present in BDNF HETs, would alter the long-term effects of chronic METH treatment on PPI. To this end, we developed a 3-week long escalating METH dosing regime, using a similar range of doses and frequency of injections to other escalating METH treatments in mice in the literature (Chao et al., 2012; Pogorelov et al., 2012). This METH treatment protocol caused significant hyperactivity during the treatment period. Unlike many previous studies of the effects of METH on PPI, behavior was examined after a 2-week break period, as we aimed to model the long term psychotic symptoms that occur in some METH users, rather than transient psychosis following METH which is probably less relevant to the pathophysiology of schizophrenia. For this reason, it is not surprising that our results differ from those of previous studies that assessed the immediate effects of METH on PPI. In experiments where PPI is tested following repeated pre-treatment with METH, this was found to disrupt PPI immediately following the final METH treatment. However METH pre-treated, non-drug challenged controls were not used in these experiments (Arai et al., 2008; Hadamitzky et al., 2011). One group has recently examined the effects of escalating METH treatment on PPI in mice after a break period, and found reduced PPI at baseline that could be rescued by electroconvulsive shock treatment (Chao et al., 2012). However there are major strain differences in PPI (Willott et al., 2003), and this study used CD-1 mice which may account for the differences in our findings. Additionally, while we calculated baseline PPI from saline challenge sessions, this previous study examined PPI without challenge injections. The acute mild stress associated with saline injection has previously been shown to reduce PPI in some mouse strains (Wang et al., 2003), as increased stress hormone levels can alter PPI (van den Buuse et al., 2004). Given these differences between previous studies and our own, this highlights the importance of strain, timing following METH treatment and behavioral testing protocol on the effects of METH in rodent studies.

Our finding of reduced sensitivity or tolerance to a challenge dose of AMPH in METH pre-treated females is in contrast to a previous study, which showed that 1 week pre-treatment with low dose METH in male CD-1 mice enhanced the effects of a sub-threshold dose of METH (1 mg/kg) to disrupt PPI (Arai et al., 2008). However, given that our effects of METH were female-specific it is unclear how this previous result compares to our finding of tolerance. Furthermore we used a higher challenge dose of AMPH (5 mg/kg), and it may be of interest for future studies to examine sub-threshold doses of AMPH in our model. Another key difference between these studies was the inclusion of a break period in our own study, whereas “sensitization” was observed immediately followed the METH pre-treatment period (Arai et al., 2008). The aim of our study was to model behavioral changes accompanying persistent psychosis following METH abuse, whereas the findings of other studies which examine behavior immediately following METH exposure may be more reflective of what occurs during transient psychosis following METH use (Sato et al., 1992). In contrast to our observation of tolerance to the effects of AMPH on PPI, in preliminary studies we have seen long-term locomotor sensitization to an AMPH challenge in this model (unpublished observations) which has also been shown previously by others following METH pre-treatment (Hall et al., 2008). This suggests that METH-induced plasticity occurring in the pathways regulating PPI is different to that which occurs in the pathways mediating locomotor sensitization. This may involve different contributions by monoamine neurotransmitters in these neural pathways.

The effects of BDNF depletion on PPI have also been examined previously. For example, in a previous study we did not observe baseline deficits in PPI in BDNF HETs (Klug et al., 2012). However, in that study, PPI was examined in animals that had not previously received chronic injections, and the response of BDNF HETs following a challenge drug may have been altered in the PPI paradigm immediately following that unfamiliar mild stressor. Forebrain restricted knockout of BDNF also resulted in no changes in baseline PPI in the absence of a challenge injection (Gorski et al., 2003). Our current observations of reduced baseline PPI in BDNF HETs irrespective of sex make this a novel model of sensorimotor gating deficits observed in schizophrenia, and future studies should aim to characterize the predictive validity of this finding by examining the response to current antipsychotic medications.

The significant effect of BDNF depletion on AMPH-disrupted PPI, but not on the effects of APO or MK-801, suggests differential changes in monoaminergic regulation of PPI pathways in BDNF HETs. Changes in the amount of dopamine release and reuptake following AMPH challenge, without changes in post-synaptic receptor expression, may explain the altered sensitivity to AMPH without major changes in the effects of the D1/D2 receptor agonist APO. Supporting this idea, dopamine levels (measured by HPLC and *in vivo* microdialysis) are increased in BDNF HETs in the caudate putamen, which is also associated with reduced dopamine transporter (DAT) function (Dluzen et al., 1999; Bosse et al., 2012). However these studies were conducted in male BDNF HETs, and further characterization of the dopaminergic system in both sexes may be necessary to understand the sex differences observed in our study. NMDA receptor-mediated regulation of PPI appears to be unaffected by BDNF depletion and METH treatment based on our findings of no changes in the sensitivity to the disruptive effects of MK-801.

It should be noted that, in addition to dopaminergic effects, changes in serotonergic activity may also play a role in altered sensitivity to the disruptive effects of AMPH on PPI in male BDNF HETs. Although the psychotropic actions of AMPH appear to be largely mediated by increasing subcortical dopamine release (Sulzer et al., 2005), one group has recently reported major contributions of both serotonergic and dopaminergic signaling in the effects of the active AMPH metabolite p-hydroxyamphetamine on PPI in mice (Onogi et al., 2010, 2011). Alterations in serotonin levels and transporter function have also been described in BDNF HETs in the CA3 region and ventral hippocampus (Daws et al., 2007; Deltheil et al., 2008; Guiard et al., 2008). The only study to assess sex differences in the serotonin system of BDNF HETs found that males, but not females, showed a deficit in serotonin transporter clearance rate in the CA3 at 2 months of age (Daws et al., 2007). Further characterization of the serotonin system in both sexes of BDNF HETs and in additional brain regions may clarify the involvement of serotonergic changes in the results that we have observed. We have previously shown that serotonergic lesions of the hippocampus in rats disrupt baseline PPI (Kusljic and van den Buuse, 2004), and it is possible that altered hippocampal serotonin system function in BDNF HETs may also contribute to changes in baseline PPI in both sexes.

Sex-differences in BDNF signaling have been reported extensively before (Wu et al., 2013), although information about sex-differences in behavior are somewhat lacking in the analysis of BDNF HETs. We previously demonstrated in male C57BL/6 mice that BDNF protein levels in the striatum and frontal cortex are correlated with serum testosterone levels during adolescent development, including a peak in BDNF protein levels at 8 weeks of age. In female mice, no such relationship was observed with serum estradiol levels (Hill et al., 2012). These findings suggest that BDNF is differentially involved in brain development during this period between the sexes, and may help to explain sex-specific changes in BDNF HETs. Specifically, if BDNF plays a differential role in the development of fronto-striatal circuits in male mice, this may result in altered monoaminergic regulation of PPI in male BDNF HETs, leaving these pathways spared in female BDNF HETs.

BDNF HETs also showed an increase in acoustic startle response. We observed increased bodyweight in these mice, which may have contributed to these effects. This has been described by others in BDNF HETs previously, and was associated with hyperphagia, hyperglycemia, insulin resistance, and hypoactivity (Lyons et al., 1999; Duan et al., 2003; Coppola and Tessarollo, 2004). In addition to these baseline startle differences, there were genotype effects on the action of AMPH and APO on startle response. Startle response in BDNF HETs was generally more affected by these drugs, with BDNF HETs being more sensitive to the effects of APO irrespective of METH pre-treatment, whereas only WT mice were insensitive to the effects of AMPH following METH treatment, and METH-treated HETs responded like saline pre-treated mice. All of these effects on startle were unaffected by sex of the animals. Therefore this is unlikely to have contributed to the changes in PPI that were observed, and in particular the changes in the sensitivity to the disruptive effects of AMPH which were sex-specific.

Our findings suggest that BDNF is not involved in the long term effects of METH treatment during young adulthood on PPI. In contrast, previous work has demonstrated that BDNF is involved in the acute neuronal response to METH and sensitization to cocaine (Narita et al., 2003; Bahi et al., 2008). These differences suggest that BDNF acts in discrete neural pathways following exposure to stimulants such as METH and cocaine, and plays a role in selective behavioral changes following these interventions. Interestingly there were large sex differences in the independent effects of both BDNF depletion and METH treatment during young adulthood. Sex differences in the role of BDNF during adolescent development of the forebrain may account for some of these effects (Hill et al., 2012). These findings highlight the need to study both sexes in preclinical research into the effects of genetic and environmental factors on behavioral endophenotypes related to schizophrenia, something which is severely lacking in the current literature on the behavioral effects of METH. The current findings warrant further investigation into the sex-differences that result following BDNF depletion and METH treatment separately, but do not exclude the value of further research into the interactions between BDNF signaling and METH-induced dysfunction in other behavioral paradigms, such as those related to the negative and cognitive symptoms of schizophrenia.

In summary, we have demonstrated that BDNF depletion and METH treatment do not have interactive effects on PPI of the acoustic startle reflex, or its disruption by AMPH, APO, or MK-801. In contrast, there were sex-specific and independent effects of these two interventions, particularly on the sensitivity to the disruptive effects of AMPH. These findings suggest that METH users may show altered regulation of PPI, and that these effects may be female-specific and are unlikely to involve altered BDNF signaling. BDNF HETs of both sexes showed baseline changes in PPI and startle reactivity, but AMPH disrupted PPI was enhanced in male mice only. BDNF HETs may be a valuable model for further research into the effects of BDNF signaling in PPI brain circuits, and particularly sex-differences, which may contribute to sensorimotor gating deficits in patients with schizophrenia. Disrupted sensorimotor gating in schizophrenia is associated with a significant level of function impairment, and better understanding of the molecular pathology in neural circuits that cause this behavioral disturbance in schizophrenia should assist in the development of more efficacious therapies.

## Materials and methods

### Animals

Male and female BDNF HETs and WT littermates were obtained from a breeding colony at the Florey Institute of Neuroscience and Mental Health. This colony was originally established with breeders from JAX Mice and Services (Bar Harbour, ME, USA) and maintained on a C57Bl/6 background. Experimental mice were weaned at 3 weeks of age and transported to the Mental Health Research Institute facility for all behavioral experiments. Mice were housed in individually ventilated cages (IVC, Tecniplast, Buguggiate, Italy) in same sex groups of 2–6 animals, with *ad libitum* access to rodent chow and tap water. The light period of the circadian cycle was from 07:00 a.m. to 07:00 p.m. and experiments were conducted during the light period. All experiments were done in accordance with the Australian Code of Practice for the Care and Use of Animals for Scientific Purposes set out by the National Health and Medical Research Council of Australia and were approved by the Florey Institute of Neuroscience and Mental Health Animal Experimentation Ethics Committee.

### Drug treatment during adolescence/early adulthood

Groups of mice in separate cages were randomly allocated to receive METH [(±)-METH-HCl, National Measurement Institute, Pymble, NSW, Australia] or saline vehicle solution (0.9% sodium chloride, Baxter Healthcare, Old Toongabbie, NSW, Australia) treatments which commenced at 6 weeks of age. Numbers in each treatment group were as follows: Male WT Saline *n* = 10, Male WT METH *n* = 9, Male BDNF HET Saline *n* = 9, Male BDNF HET METH *n* = 9, Female WT Saline *n* = 10, Female WT METH *n* = 10, Female BDNF HET Saline *n* = 8, Female BDNF HET METH *n* = 8. Treatments were administered by intraperitoneal (IP) injection in the mouse housing room following an escalating dosing regime that has previously been used to mimic human patterns of abuse [Robinson and Camp (1987), Figure 1A shows treatment regime in experimental timeline]. During the first week of treatment, animals received one daily injection of 1 mg/kg METH in the morning (between 8 and 10 a.m) from Monday to Friday. During the second week of treatment, animals received two daily injections of 2 mg/kg METH, in the morning and afternoon (between 4 and 6 p.m.) from Monday to Friday. In the third week of treatment, animals received two daily injections of 4 mg/kg METH, in the morning and afternoon from Monday to Friday. Every Friday, the acute response of the animals to METH treatment was assessed in locomotor photocell cages. Vehicle injections matched the frequency of METH treatments throughout this 3-week period. In all cases, injection volumes of 10 ml/kg were administered using 30 g needles, and no injections were administered on weekends. Following the end of the treatment period, mice were left undisturbed for 2 weeks before commencement of PPI testing.

### Behavioral testing during treatment: locomotor activity

To measure the sensitivity of animals to METH administration during the treatment period, locomotor activity was measured immediately following treatment administration on Friday mornings during the 3 week treatment period. Locomotor photocell arenas [27 × 27 × 40 cm (l × w × h), TruScan, Coulbourn Instruments, Whitehall, PA, USA] were used to measure locomotor activity. Mice were habituated to the testing arena during two 1 h sessions in the 2 days prior to the first testing session. On Friday mornings during the treatment period, mice received their injections in the locomotor testing room and were immediately placed into photocell arenas. Horizontal activity was recorded for 1 h and was expressed as locomotor distance moved (cm) per 5 min interval.

### Behavioral testing during adulthood: prepulse inhibition

Baseline and psychostimulant-induced disruption of PPI was assessed in mice using a pseudorandomized repeated-measures paradigm where mice were tested using all challenge drugs with 3–4 days washout between sessions. These drugs were chosen because they all disrupt PPI albeit via a differential pharmacological mechanism of action, i.e., acute dopamine release (AMPH), non-selective dopamine receptor agonism without effects on dopamine release (APO) and a non-dopaminergic mechanism involving NMDA receptor antagonism (MK-801) (Geyer et al., 2001; van den Buuse, 2010). AMPH (d-amphetamine sulfate, 5 mg/kg, Sigma, St. Louis, MO, USA), APO [R-(-)-APO hydrochloride hemihydrate, 5 mg/kg, Sigma] and MK-801 [(+)-MK-801 hydrogen maleate, 0.25 mg/kg, Sigma] were used as challenge drugs in the current study, and these doses have been used previously to produce a robust disruption of PPI in mice (Chavez et al., 2009). These were dissolved in saline, with saline administered as the control solution. All drugs were administered IP 10 min before the start of the PPI session.

All mice were tested for startle, startle habituation and PPI of startle using previously published protocols (van den Buuse et al., 2009). Briefly, we used San Diego Instruments (San Diego, CA, USA) SRLab automated startle boxes and the PPI session consisted of 104 trials, taking approximately 36 min to complete. The protocol commenced with 3 min of 65 dB background noise. The session started and ended with a block of 8 trials delivering only a 115 dB pulse. Together with 16 pseudorandomly delivered pulse-alone stimuli during the main component of the session, responses to these startle pulses were used to construct startle habituation curves. PP-pulse trials consisted of a 115 dB pulse, preceded either by 30 or 100 ms by a PP of 2, 4, 8, or 16 dB over the 65 dB background noise (total SPL 67, 69, 73, or 81 dB). PPI was calculated as the difference between the median response to PP-pulse trials and the median response to pulse-alone trials, expressed as a percentage of the median response to the pulse-alone trials. The session also included 8 trials during which no sound stimulus was delivered, in order to check for non-specific movements.

### Statistical analyses

All data were expressed as mean ± standard error of the mean (SEM). Differences within and between groups were analyzed by analysis of variance (ANOVA) with repeated measures (Systat, version 9, SPSS Inc., USA). For analysis of PPI data, PP intensity, startle block, ISI and acute drug challenge were included as within-group, repeated measures factors, while METH treatment, genotype, and sex were included as between-group factors. The effect of a drug challenge to disrupt PPI was considered significantly different when there was a drug challenge × between group factor interaction, using a repeated measures ANOVA that included data from saline and drug challenge sessions. For analysis of locomotor activity during the treatment period, week of treatment period and time during session were included as within-group, repeated measures factors, while METH treatment, genotype, and sex were included as between-group factors. When *p* < 0.05, differences were considered statistically significant. The Greenhouse-Geisser Epsilon correction was applied when multiple repeated measures were compared in the same ANOVA test.

### Conflict of interest statement

The authors declare that the research was conducted in the absence of any commercial or financial relationships that could be construed as a potential conflict of interest.
